# Multilayer radiation shielding system with advanced composites containing heavy metal oxide nanoparticles: a free-lead solution

**DOI:** 10.1038/s41598-023-45621-2

**Published:** 2023-10-27

**Authors:** Wafa M. Al-Saleh, Haifa M. Almutairi, M. I. Sayyed, Mohamed Elsafi

**Affiliations:** 1 College of Science and Health Professions, King Saud Bin Abdulaziz University for Health Sciences, P.O. Box 6664, 31982 Hofuf, Al-Ahsa Saudi Arabia; 2 King Abdullah International Medical Research Center, Hofuf, Al-Ahsa Saudi Arabia; 3 Department of Physics, Faculty of Sciences, Umm AL-Qura University, 24382 Mecca, Saudi Arabia; 4 Department of Physics, Faculty of Science, Isra University, Amman, Jordan; 5 Renewable Energy and Environmental Technology Cente, University of Tabuk, Tabuk, 47913 Tabuk, Saudi Arabia; 6 Physics Department, Faculty of Science, Alexandria University, Alexandria, 21511 Egypt

**Keywords:** Materials science, Physics

## Abstract

With the use of multilayer materials such as concrete, mortar and ceramics that were fortified with PbO, WO_3_ and Bi_2_O_3_ nanoparticles, our study's objective was to produce a an effective photon shielding system. Experimental evaluation of the radiation shielding efficiency of two sets of samples with various thicknesses was conducted. The elemental content and morphology of the samples were corroborated by SEM and EDX studies, with ceramic samples exhibiting superior particle distribution and fewer voids than concrete and mortar specimens. The linear attenuation coefficient (LAC) was studied both experimentally and numerically using the Phy-X program, and it was found that the two sets of values were in satisfactory agreement. The values of LAC were consistently greater for samples with 30% of the selected heavy metal oxides than for those with 10%. The LAC for Cer-1 was 5.003 cm^−1^ at 0.059 MeV, whereas the corresponding LAC for Cer-2 was 2.123 cm^−1^. The LAC values were as follows: ceramics (5.003 cm^−1^), mortar (2.999 cm^−1^), concrete (2.733 cm^−1^), and the transmission factor (TF) examination of the multiple-layer specimens showed that the TF of the 3 cm thick multilayer sample was lower than that of the 2 cm thick sample and that both multilayer samples displayed better attenuation efficiency in comparison to single-layer specimens. The results show the possibility for employing multilayer structures with different densities, thicknesses, and sizes in suitable radiation shielding applications.

## Introduction

With the progression of society and advancements in technology, there are several applications for ionizing radiation, including the non-destructive industries, medical research, nuclear power generation, and scientific research geological investigation. As a direct consequence of this, the total number of gamma radiation tests performed each year continues to rise. Radiation exposure from medical sources represents for more than 80% of the total radiation exposure; therefore, it is a natural worry on a global scale. Erythema, which is an immediate result of radiation exposure, as well as genetic impact, that can induce cell mutation and cancer, are both potential risks associated with radiation^1–4^. Accordingly, radiation shields are required in order to provide adequate protection from the deleterious effects of ionizing radiation. In order to attain protection from radiation, several different kinds of materials and compounds have been employed^5–9^. These days, shielding materials that are typically utilized for nuclear and medical facilities are frequently consisting of heavy metal components, and neutron absorbers. Additionally, a successful shield is often composed of multiple layers. In order to properly protect against a wide variety of kinds of ionizing radiation, multi-layer shielding often entails using a combination of materials that each have their own unique set of features. The materials that are utilized can be different based on the application, and the thickness for every layer is also a significant issue^10–13^. In general, a layer that is thicker will offer higher protection. It is possible for multiple layers of radiation shielding to give better adequate protection from ionizing radiation. This is achieved by increasing the quantity of material which the photons must pass across, which in turn lowers the intensity of the photons. This strategy has the potential to offer supplementary protection against scattered radiation as well as secondary radiation that is generated when radiation travels via the shields^1, 14^.

For instance, during diagnostic imaging operations, a multi-layer shield is frequently utilized in healthcare facilities to provide protection from X-rays that are produced. This may involve a layer of lead, which would absorb high-energy X-rays, then by a layer of concrete, which would collect scattered radiation, and then a layer of drywall, which would absorb any low-energy radiation that was left over. Furthermore, it is common practice in nuclear facilities to employ many layers of shielding in order to protect personnel and the surrounding environment from the potentially hazardous impacts of ionizing radiation caused by nuclear reactions. This may involve the use of many layers of concrete and steel in order to absorb gamma radiation and neutrons and so lower their intensity^15, 16^.

Nanomaterials have the possibility of playing a function in multi-layer radiation shielding by providing novel materials and methods for improving the efficiency of current shielding materials^17–19^. The usage of nanocomposite materials, which mix conventional shielding components with nanoscale particles to produce new hybrid composites with increased radiation shielding capabilities, has been investigated by a variety of researchers. These materials have the ability supply better levels of safety since they are capable of absorbing a greater proportion of the radiation and lowering its intensity in a more efficient manner^20–22^. Nanoparticles made of bismuth oxide (Bi_2_O_3_), tungsten trioxide (WO_3_), and lead oxide (PbO) are utilized in the radiation shielding process. It has been demonstrated that these three types of nanoparticles have excellent radiation-absorbing characteristics and are able to efficiently decrease the intensity of gamma radiation. It is feasible to develop multi-layer radiation shielding systems by employing these nanoparticles in conjunction with other shielding materials. These systems offer better levels of safety and increased performance in comparison to typical shielding materials.

In experimental studies of the radiation shielding characteristics of multi-layer radiation shielding systems, there are a variety of methodologies that may be used to evaluate the efficiency of the shielding in order to determine whether or not the shielding is beneficial. Utilizing radiation sources (i.e. different radioactive point sources), to generate gamma photons that can be detected both before and after it has passed through the shielding materials is a typical method. It is possible to assess the efficacy of the shielding by calculating the amount by which the intensity of the radiation is reduced after it has passed through the shielding. So, in this work, we prepared novel multi-layer radiation shielding systems containing Bi_2_O_3_, WO_3_ and PbO nanoparticles. We used an experimental method to estimate the radiation shielding performance of these new shielding systems.

## Material and methods

**Figure 1:**
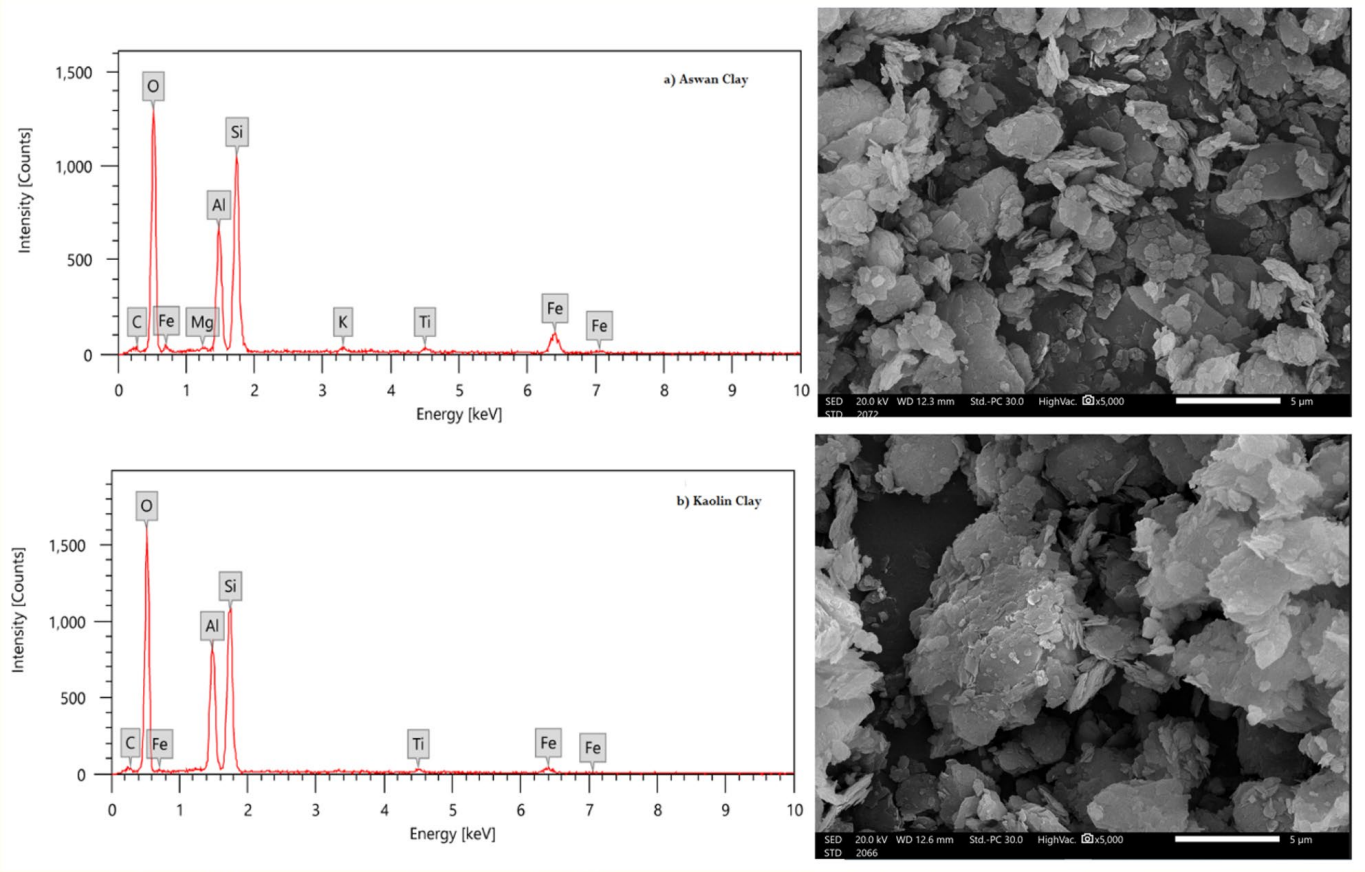
SEM and EDX analysis of raw materials in ceramic preparation, (**a**) EDX of Aswan clay, (**a′**) SEM image of Aswan clay, (**b**) EDX of kaolin clay, (**b′**) SEM image of kaolin clay.

**Figure 2:**
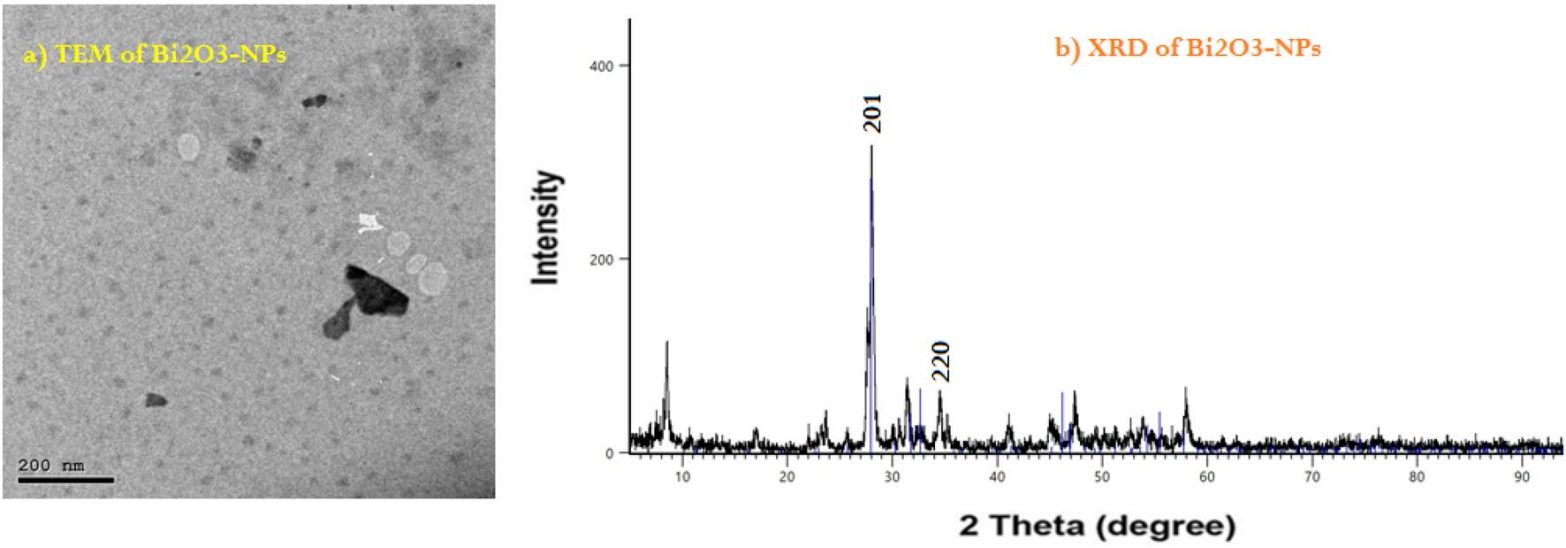
TEM and XRD analysis of Bi_2_O_3_ nanoparticles, (**a**) TEM of Bi_2_O_3_-NPs, (**b**) XRD of Bi_2_O_3_-NPs.

The propose of this study to form a perfect wall against photons (X- and Gamma-rays) from multilayer basic materials such as concrete, mortar and ceramic. In this study we prepared two different samples with different thicknesses from Ceramics, Concrete and Mortar. The raw materials of present ceramics were kaolin clay, Aswan clay, and bismuth oxide, where the first ceramic composite was Cer.1: 35% Aswan clay + 35% kaolin clay + 30% Bi_2_O_3_ (by weight) and the second type was Cer.2: 45% Aswan clay + 45% kaolin clay + 10% Bi_2_O_3_ NPs. The EDX analyses “Energy Dispersive X-ray”^23^ of these raw materials as well as the SEM images “Scanning electron microscope”^24^ were done before the preparation as shown in Fig. 1. From the SEM images in Fig. 1, the size of the minerals in the present clay was measured, where the average size was 5 μm and the shape and relationships between phases such as coating or crystal erosion were observed. From the EDX analysis, the elemental percentages of both clays are determined and reported in Table 1. To determine the particle size of Bi_2_O_3_ NPs, the TEM-image “Transmission electron microscope” and the XRD analysis “X-Ray Diffraction”^25^ were determined in Fig. 2. The morphology results confirmed that Bi_2_O_3_-NPs are circular spots with an average diameter ranging from 10 to 30 nm. This helps in better distribution within the mixture between the molecules, which improves the shielding properties of the composites. The current ceramics were prepared and composed with the same idea as the previous literature^26^, where the components were mixed using a ball mill until it became homogeneous, then the mixture was stirred with water to obtain a paste and placed in a crucible and fired in electric furnace at 1200 °C.

**Table 1:** The elemental compositions of Aswan and Kaolin clay obtained from EDX-analysis.

Element	Elemental mass (%)	
	Aswan clay	Kaolin clay
C	2.26 ± 0.18	3.65 ± 0.21
O	53.50 ± 0.56	57.92 ± 0.55
Mg	0.29 ± 0.06	–
Al	12.63 ± 0.25	14.18 ± 0.25
Si	21.42 ± 0.34	21.10 ± 0.32
K	0.65 ± 0.07	–
Ti	1.00 ± 0.09	0.86 ± 0.08
Fe	8.26 ± 0.29	2.29 ± 0.15
Total	100.00	100.00

**Figure 3:**
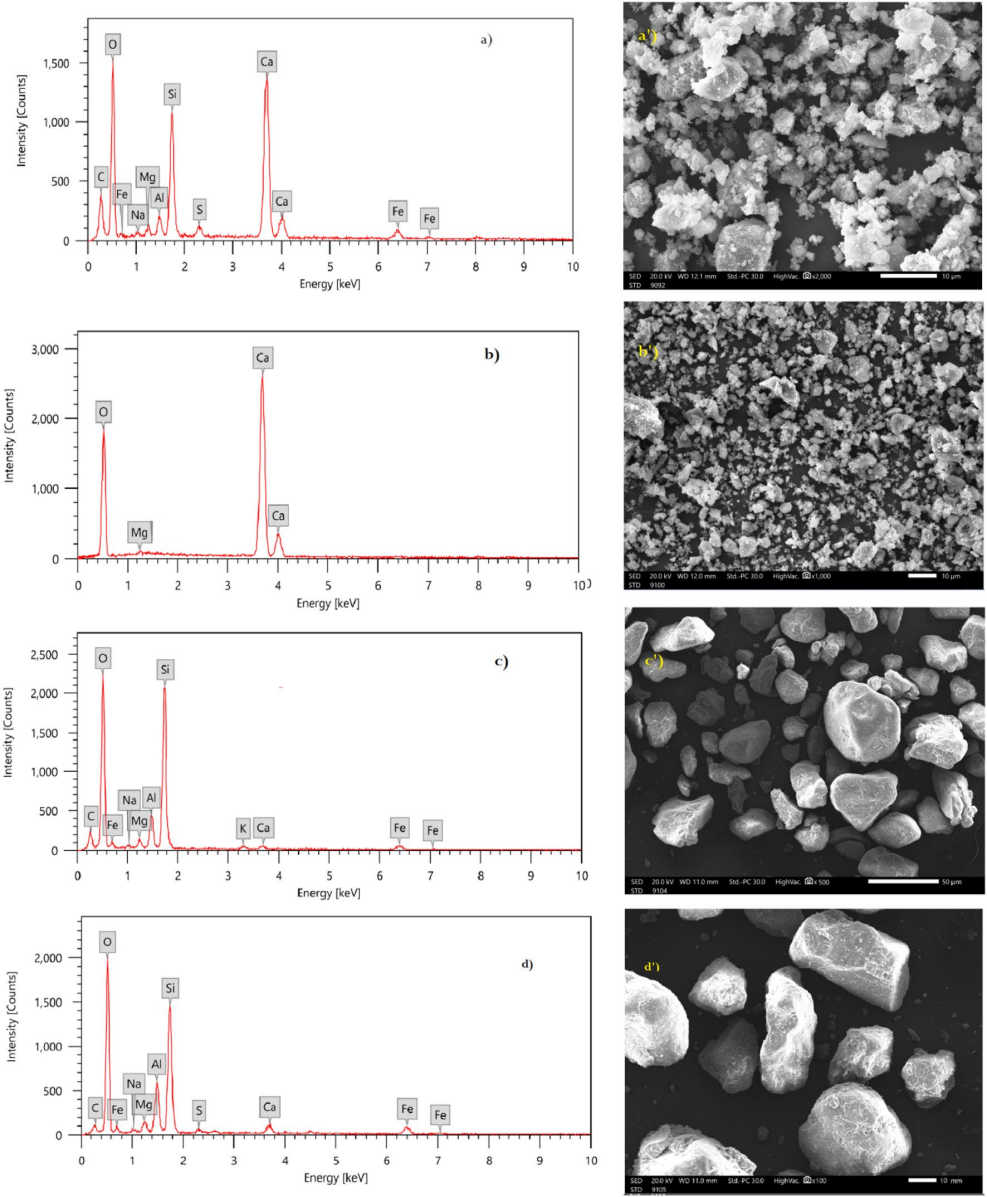
SEM and EDX analysis of raw materials in mortar and concrete preparation, (**a**) EDX of cement, (**a**′) SEM image of cement, (**b**) EDX of hydrated lime, (**b**′) SEM image of hydrated lime, (**c**) EDX of sand, (**c**′) SEM image of sand, (**d**) EDX of gravel, (**d′**) SEM image of gravel.

**Figure 4:**
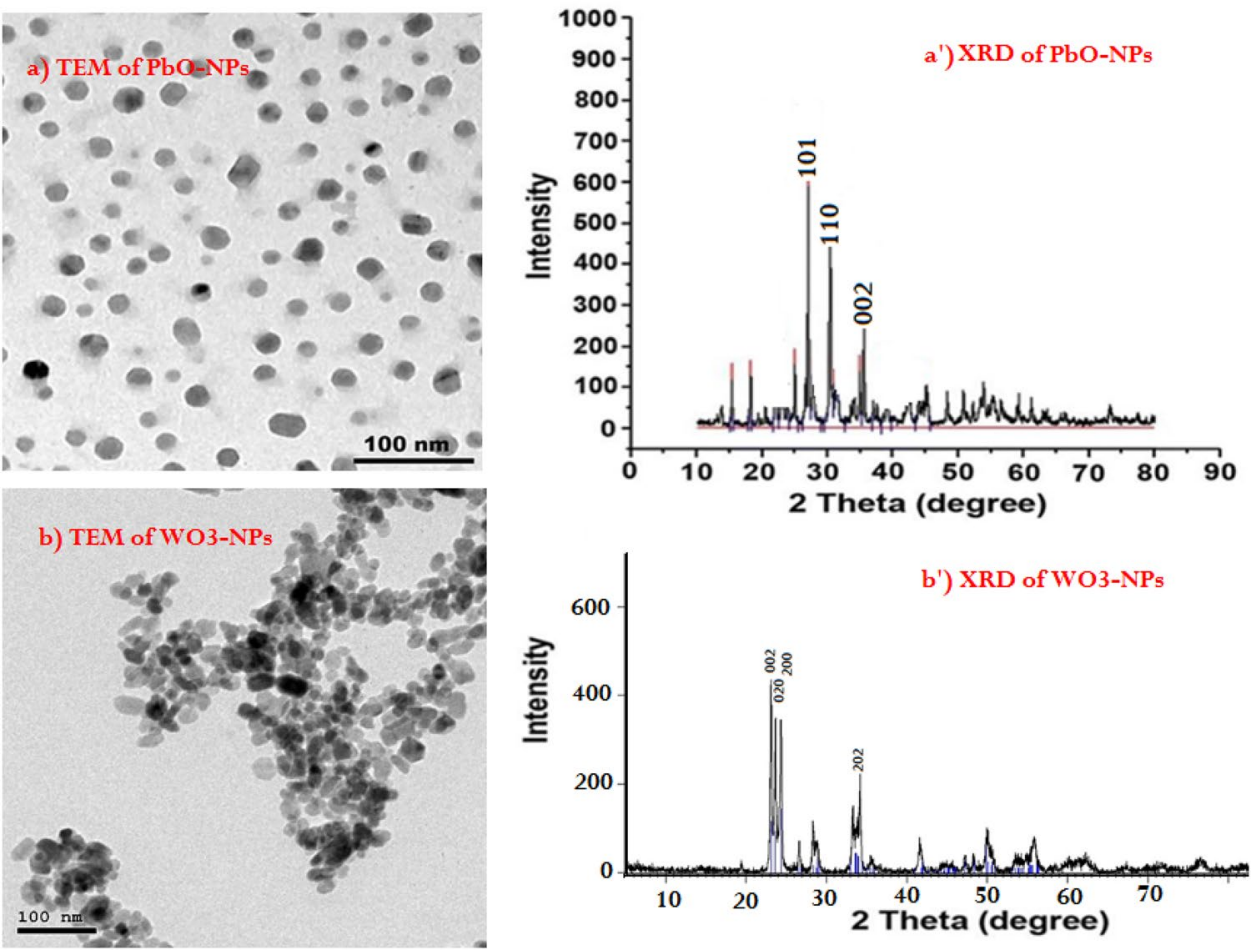
TEM and XRD analysis of PbO and WO_3_ nanoparticles, (**a**) TEM of PbO-NPs, (**a**′) XRD of PbO-NPs, (**b**) TEM of WO_3_-NPs, (**b**′) XRD of WO_3_-NPs.

The mortar and concrete were prepared traditionally based on previous literatures^27^. The compositions are presented in Table 2, since in both preparations the heavy metal oxides (PbO and WO_3_) were putted as a partial replacement of sand, where in Mor.1 and Conc.1 mixtures, the 30% of PbO and WO_3_ replacement, respectively, while in Mor.2 and Conc.2 mixtures, the 10% of PbO-NPs and WO_3_- NPs replacement happened, respectively. Before the preparation, the EDX and SEM characteristics of mortar and concrete raw components (Cement, Hydrated lime, Sand and gravels) were analyzed and determined as shown in Fig. 3 and Table 3, through SEM images of raw materials, it was able to determine the shape of each material as shown in the figure and the average size of its particles in addition to porosity. where the cement and hydrated lime are much less porous than sand and gravel. As well as the TEM and XRD analysis were done for nanoparticles used in mortar and concrete preparation (PbO-NPs and WO_3_-NPs) as shown in Fig. 4.

**Table 2:** The compositions of prepared mortar and concrete.

Materials	Mass percentage (g)			
	Mor.1	Mor.2	Conc.1	Conc.2
Cement	100.00	100.00	100.00	100.00
Hydrated lime	35.00	35.00	–	–
Sand	280.00	360.00	154.00	198.00
Gravel	–	–	440.00	440.00
PbO	120.00	–	–	–
PbO-NPs	–	40.00	–	–
WO_3_	–	–	66.00	–
WO_3_-NPs	–	–	–	22.00
(w/c) ratio	0.51	0.49	0.47	0.46

**Table 3:** The elemental compositions of cement, sand and gravel estimated from EDX-analysis.

Element	Mass, %		
	Cement	Sand	Gravel
C	4.05 ± 0.18	2.66 ± 0.22	2.86 ± 0.12
O	53.11 ± 0.53	50.57 ± 0.49	53.57 ± 0.42
Na	0.48 ± 0.05	0.67 ± 0.07	1.67 ± 0.17
Mg	1.69 ± 0.05	1.46 ± 0.08	0.46 ± 0.02
Al	2.30 ± 0.06	3.90 ± 0.15	5.90 ± 0.14
Si	10.07 ± 0.13	31.92 ± 0.24	32.92 ± 0.24
K	–	1.26 ± 0.07	–
S	0.77 ± 0.04	0.61 ± 0.05	0.61 ± 0.15
Ca	25.11 ± 0.21	1.82 ± 0.09	0.82 ± 0.03
Fe	2.41 ± 0.11	4.11 ± 0.16	1.11 ± 0.11

After preparing the six composites with different thicknesses, the morphological and mechanical properties of each mixture were studied, as the samples were photographed with an electron microscope to clarify the particle distribution within the mixture (SEM-image), and the compressive strength of the mixtures prepared from ceramics and mortar was studied using the squeeze-flow method^28, 29^, where the stress–strain curve was determined through the test till the sample crumbles from applied pressure. The thickness of the sample was 1 cm and its diameter was 8.2 cm, and the pressure was applied at a rate of 0.1 mm s^−1^.

**Figure 5:**
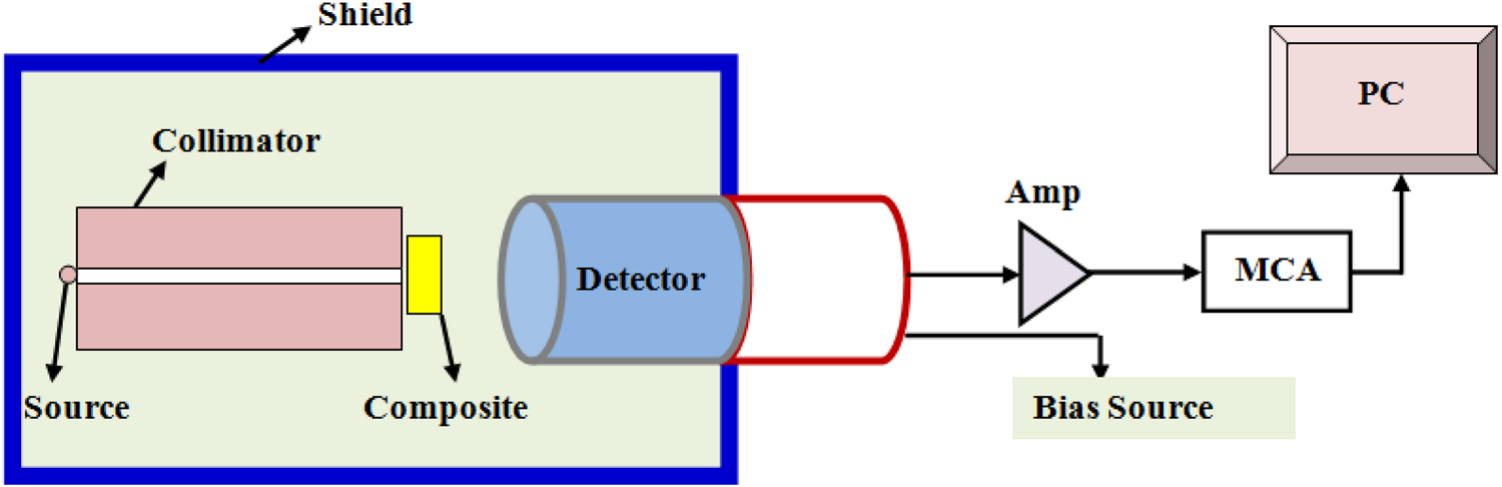
The experimental geometry in the current study.

Moreover, the shielding properties of these mixtures were determined individually (one layer) and multi-layers, where the linear attenuation coefficient (LAC) was set experimentally using a semiconductor detector (HPGe) with high efficiency and resolution^30^ and radioactive point sources such as cesium-137, cobalt-60 and americium-241^30^. The measurement was done according to the geometry shown in Fig. 5, where the measurement was done once in the presence of the sample (whether it was a layer or multi-layers) and once in the absence of the sample to obtain the photon intensity (count rate) in the presence of layer (I) and the absence of layer (I_0_), and from these values. The lead collimator was used between the source and the absorber with inner diameter 8 mm and length 10 cm to get the narrow beam experiment, without incident narrow beam, the buildup factor must be added in Eq. (1). The procedure used for multi-layer measurements is the same as the single-layer measurement procedure, only a single-layer is removed and the multi-layer are added. The LAC is calculated by the following law^31^:

1 $$LAC = \frac{1}{x}\ln \frac{{I_{0} }}{I }$$

where, x, represent the thickness of multi-layer. The values of micro-composites LAC were calculated theoretically by Phy-X software and compared with the experimental results to verify the validity of the presented values. The other essential attenuator factors such as HVL, TVL and radiation shielding efficiency (RSE) which discussed in^32^ and can be expressed by the following law:

2 $$HVL = \frac{Ln \left( 2 \right)}{{LAC}}$$

3 $$TVL = \frac{{Ln \left( {10} \right)}}{LAC}$$

4 $$RSE, \% = \left[ {1 - \frac{I}{{I_{0} }}} \right] \times 100$$

## Results and discussion

**Figure 6:**
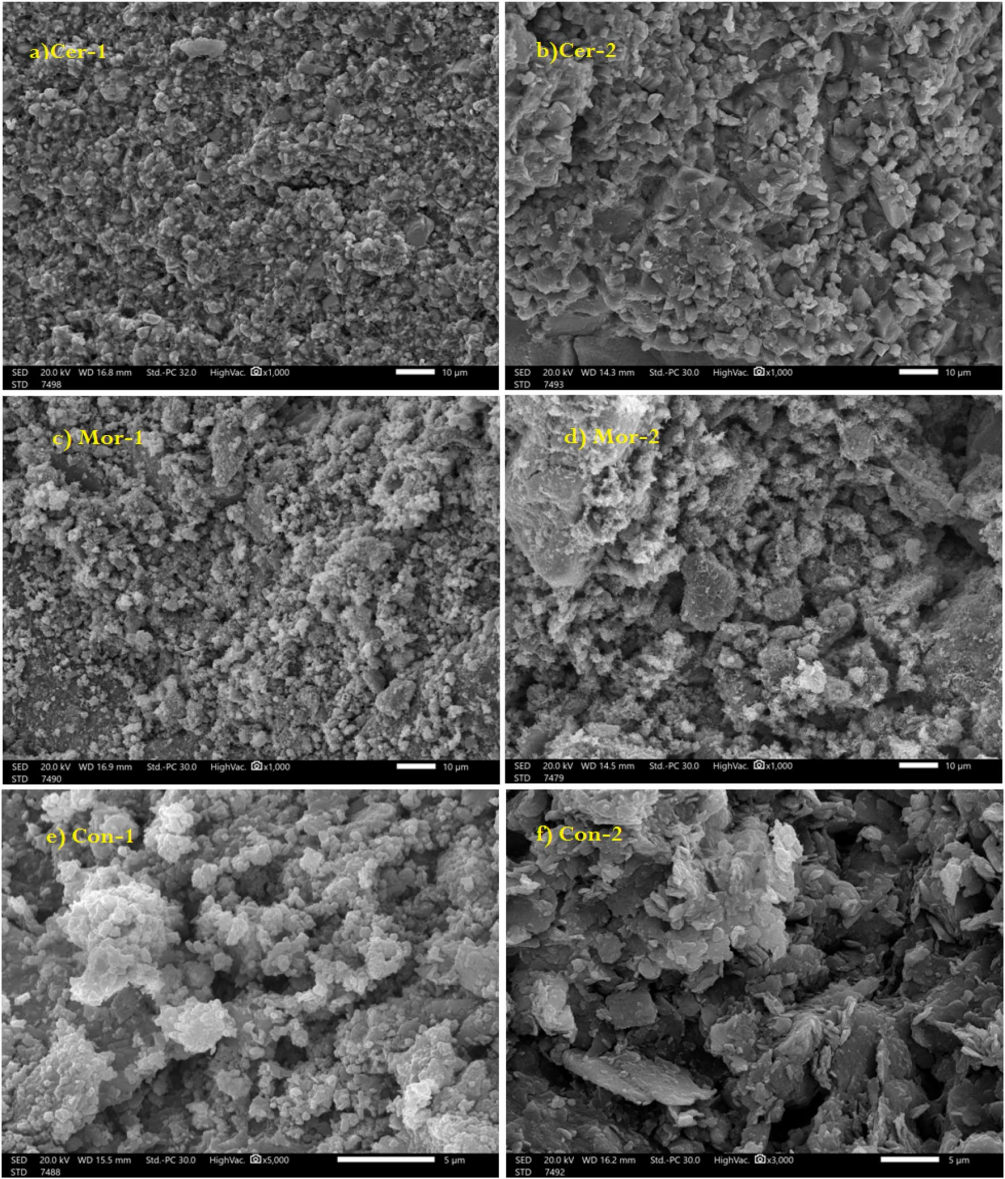
SEM images of prepared composites, (**a**) Cer-1, (**b**) Cer-2, (**c**) Mor-1, (**d**) Mor-2, (**e**) Con-1 and (**f**) Con-2.

The density of all prepared different composites was calculated with different samples and the average was taken and we found that the densities were 3.054 ± 0.006, 2.582 ± 0.012, 2.999 ± 0.010, 2.515 ± 0.007, 2.733 ± 0.021 and 2.505 ± 0.008 g cm^−3^ for Cer-1, Cer-2, Mor-1, Mor-2, Con-1 and Con-2, respectively. The increasing of density in the mixtures due to the adding of heavy metal oxides (HMO) and its clear that the highest density was for the ceramic Cer-1, while the lowest density was prepared for the concrete Con-2. After sample preparation, SEM-image were taken under x-ray accelerator voltage 20 keV, the images were represented in Fig. 6. The SEM results showed that the ceramic particles had a better distribution than mortars and concrete and fewer voids formed in the ceramic, while the largest voids were in concrete samples, in addition, more of heavy particle oxides lead to more distribution inside the mixture especially with nanoparticles of HMO^33, 34^.

**Figure 7:**
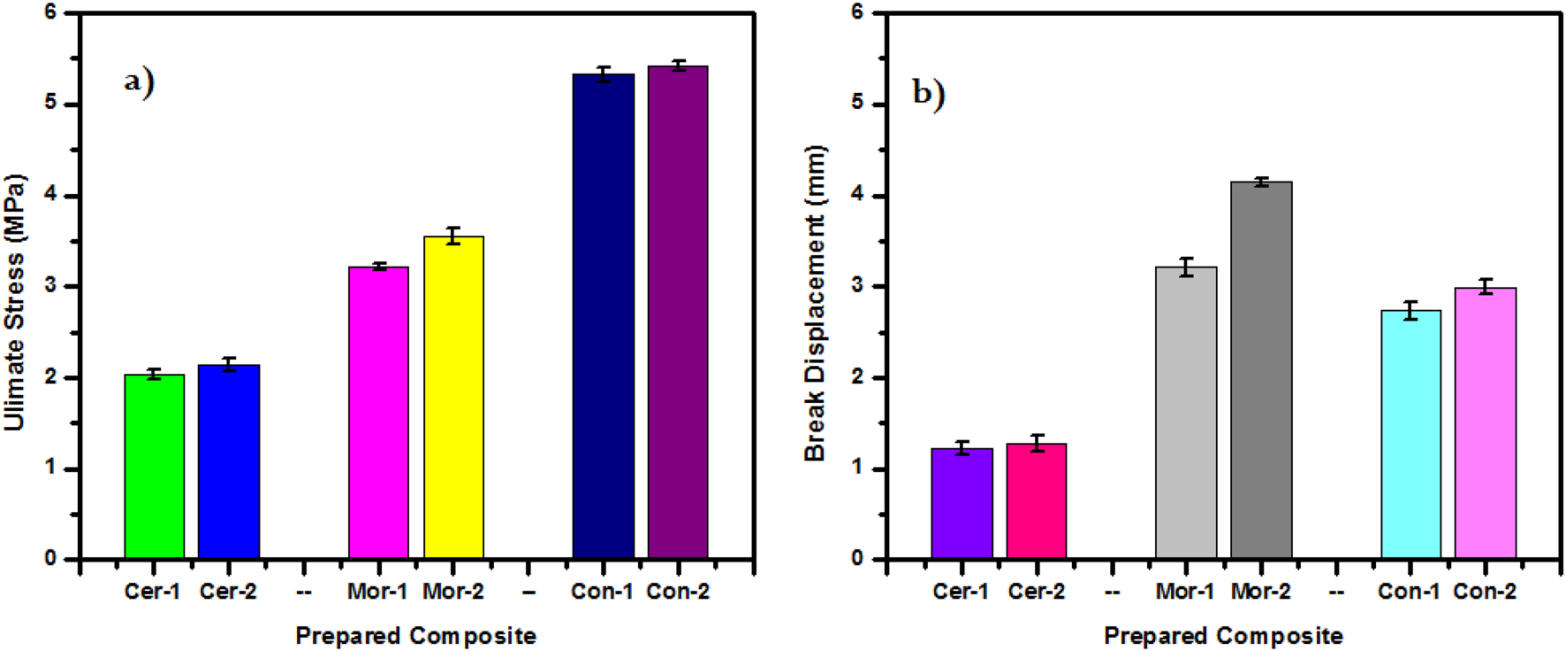
The mechanical properties of prepared mixes, (**a**) ultimate strength and (**b**) break displacement.

The mechanical properties of the squeeze type were conducted on the six different samples with the same thicknesses and conditions (two samples of each type) using the squeeze-flow technique, through which the largest stress “Ultimate Strength” required to fracture the sample was obtained after a certain displacement as a result of pressure, this displacement at which the sample was fractured or cracked It’s called “Break Displacement”^35^. The results are presented in Fig. 7, and it is clear from the results that in all mixtures, whether it is ceramics, mortar, or concrete, that the composites which contain a lower percentage of HMO (nano-HMO) are the ones that require higher force and displacement to break it, compared to samples that contain 30% HMO (micro-HMO), where the Ultimate Strength of Cer-1 and Cer-2 were 2.03 and 2.14 MPa, as well as the break displacement are 1.22 and 1.27 mm, respectively, and the Ultimate Strength of Mer-1 and Mer-2 were 3.21 and 3.55 MPa, as well as the break displacement are 3.21 and 4.14 mm, respectively, the Ultimate Strength of Con-1 and Con-2 were 5.33 and 5.39 MPa, as well as the break displacement are 2.73 and 2.99 mm, respectively. From these results, it is better to use mixtures that have better mechanical properties when using these samples as multi-layers to form a distinctive wall against ionizing radiation, and this was the reason for using Nano-HMO, as a little of it as an a filler improves the shielding properties of the absorbent material.

**Figure 8:**
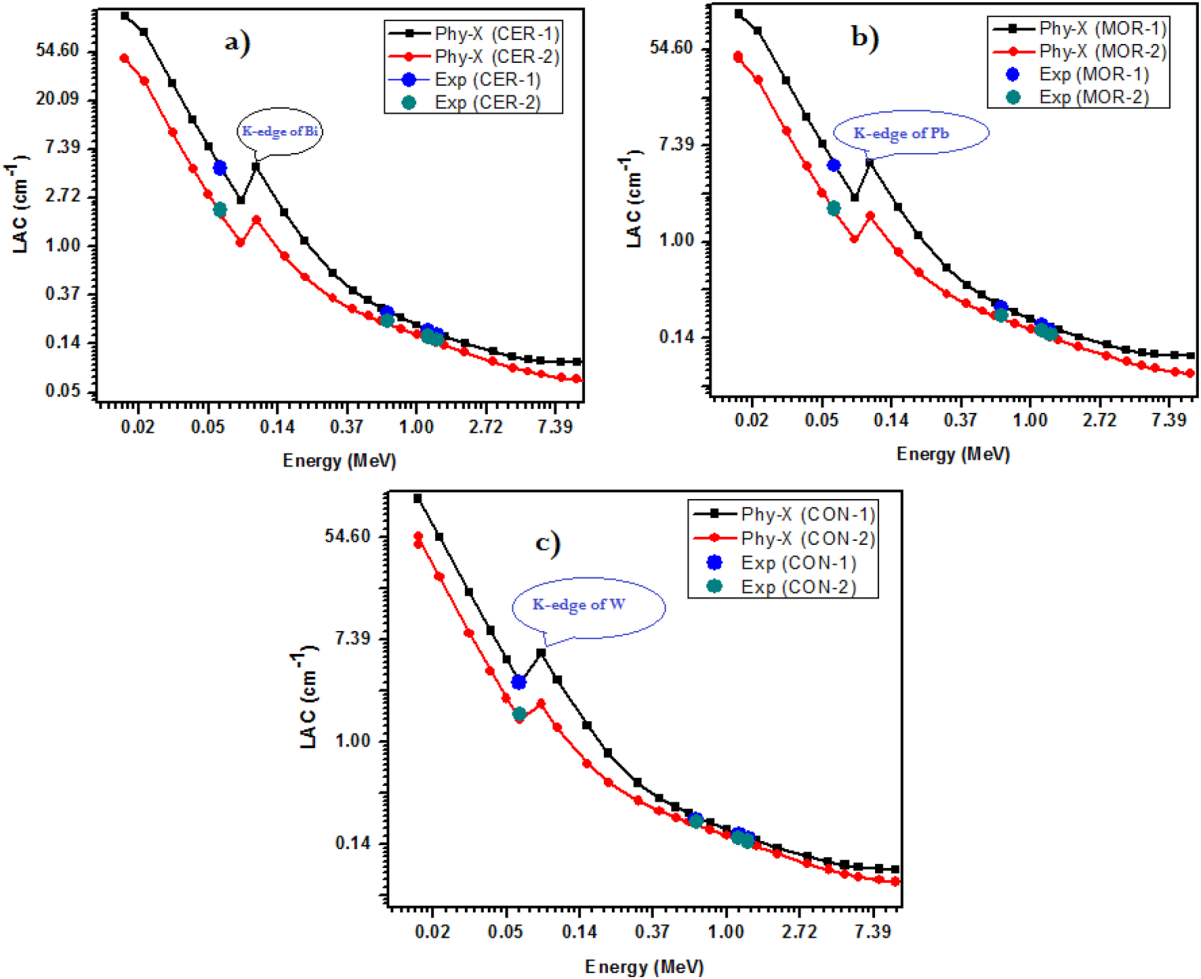
LAC of the prepared samples (**a**) for ceramic composites, (**b**) for mortar composites and (**c**) for concrete composites.

In terms of radiation shielding properties, in the first step of the attenuation study, we investigated the linear attenuation coefficient (LAC). While the direct measurements of the LAC for the prepared samples were limited to just four discrete energies, it was important to gain a broader understanding of their attenuation behavior across a wide energy range. To achieve this, we utilized the Phy-X software to calculate the LAC for three prepared samples at energies ranging from 0.015 to 15 MeV. By doing this, we were capable of to characterize their attenuation characteristics more thoroughly and accurately, which helped us decide more carefully regarding their possible uses. We conducted a comparison between the LAC collected through experiments as well as those determined with Phy-X program to guarantee the accuracy and reliability of our Phy-X findings. Three different samples were used in this evaluation, and we labeled these samples as CER-30%, MOR-30%, and CON-30%. The results for these samples are given in Fig. 8a–c respectively. This comparison played a key role in establishing the dependability and potency of our simulation approaches in predicting LAC for these specific specimens. We succeeded to acquire a more in-depth and precise comprehension of the attenuation characteristics of these materials by utilizing a combination of experimental and computational methodologies. Apparently, the experimental data are quite near to being matched by the results that were acquired by Phy-X for the LAC of the three samples. This strong indication of the precision and dependability of both approaches further confirms the use of Phy-X results as a potent tool for estimating the attenuation behavior of these types of materials. The matched data points show that our simulation methods can properly represent the samples’ attenuation properties even when obtaining direct experimental measurements may be challenging or time-consuming. It should be noted that the LAC drops dramatically with increasing energy, based on the Lambert–Beer rule. Our Phy-X results, which demonstrated a constant drop in LAC as the radiation’s energy increased, confirmed this tendency. Our LAC calculations’ conformity with the Lambert–Beer law is another evidence of their precision and dependability. We observed that the LAC of our samples had a maximum value at 0.015 MeV, followed by a sharp decline as the energy increased. The high LAC values at low energy suggested that these composites have good attenuation properties at low energy region^36^. This tendency, which is explained by the increased chance of photoelectric absorption at lower energies and a subsequent drop in this likelihood at higher energies, was consistent with other findings. As the energy increases, the reduction in LAC suggested that the attenuation performance of these composites decreases and more high energetic photons can penetrate the samples^37^.

**Figure 9:**
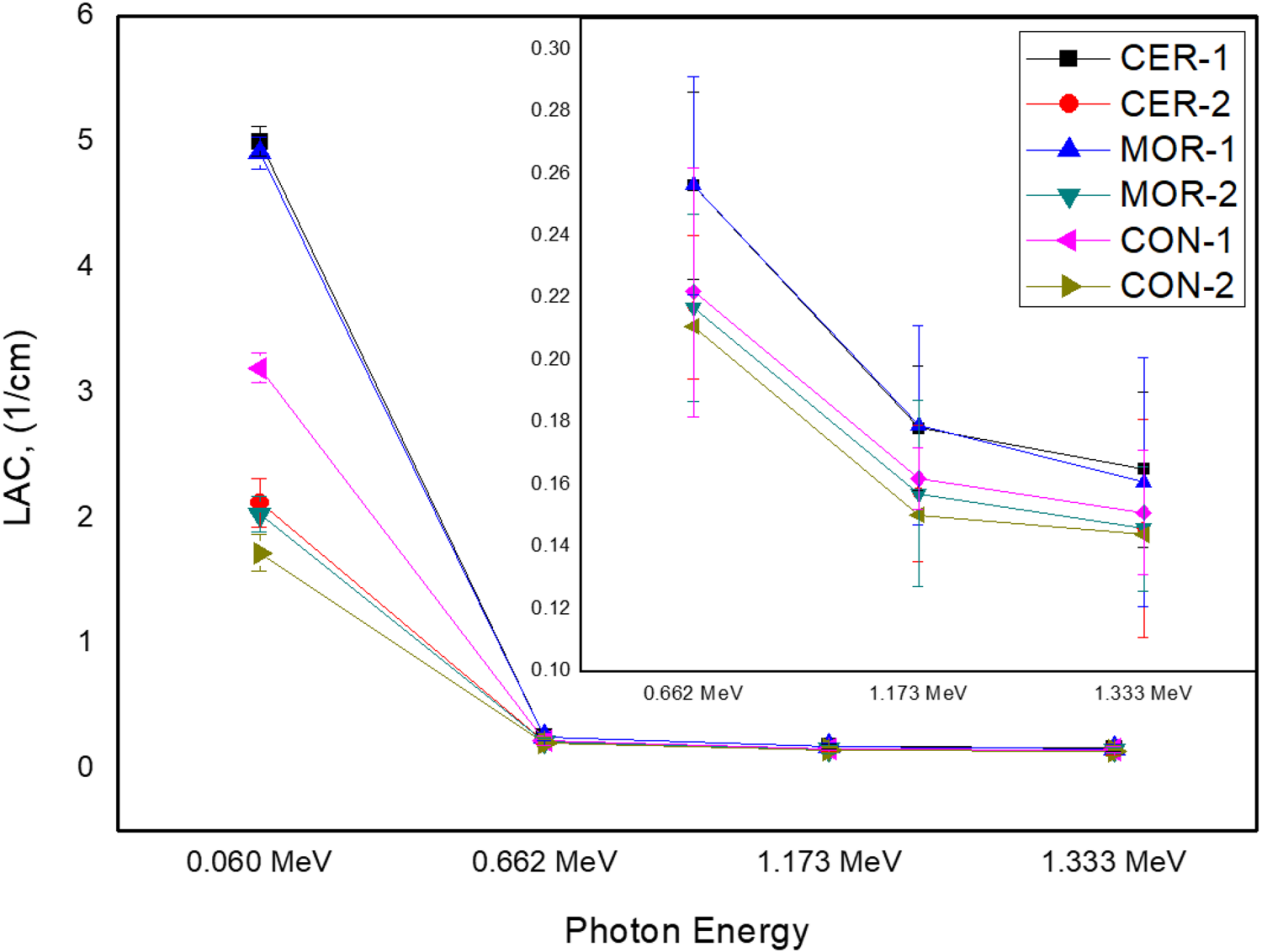
The experimental LAC of all prepared composites at different energies with the uncertainty.

**Figure 10:**
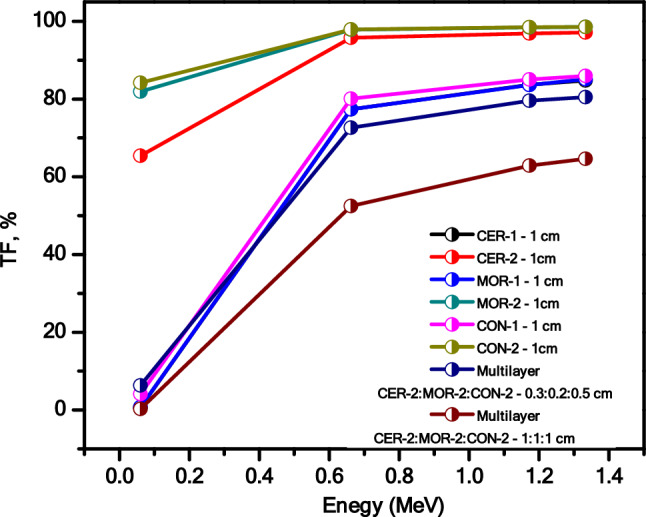
The transmission factor (TF) of all prepared composites as well as different multi-layers at different energies.

In Fig. 9, we showed the LAC for all prepared composites with its uncertainty. The uncertainty was calculated due to the uncertainty in the net count, density and thickness of the composite. We observed that the LAC for the samples which contain 30% of any of the chosen oxides (i.e. PbO, WO_3_ and Bi_2_O_3_) is higher than the samples composed of 10% of the chosen oxides. The higher LAC values in the composites with 30% of PbO, WO_3_ and Bi_2_O_3_ can be ascribed to the denser atomic packing and increased interaction of these oxides with the incoming photons. A higher amount of these compounds might lead to more shielding within the composites, resulting in a more pronounced photons absorption compared to composites with 10% of these compounds^38^. The LAC for Cer-1 is higher than the LAC for Cer-2. These results emphasize the importance of employing significant amounts of PbO, WO_3_ and Bi_2_O_3_ in order to improve the prepared samples' LAC. The additional finding from Fig. 10 is that the LAC follows the sequence LAC-Cer1 > LAC-Mor-1 > LAC-Con-1 when the LAC values for the three materials (concrete, mortar, and ceramic) were compared at a fixed amount of HMO.

We investigated the transmission factor (TF) for the prepared composites. But, we have developed prepared composites and synthesized two new samples, both of which consist of multiple layers. The first sample includes Cer-2, Mor-2, and Con-2 with thicknesses of 0.2 cm, 0.3 cm, and 0.5 cm, respectively. The second sample also includes Cer-2, Mor-2, and Con-2, but the thicknesses are 1 cm for each layer. The results indicate that the TF of the sample with a multilayer and a thickness of 3 cm is lower than the TF of the sample with the same multilayer and a thickness of 2 cm. The reported decrease in the TF for the multilayer composites with a thickness of 3 cm in comparison to the sample with thickness of 2 cm can be ascribed to the path length of the photons through the prepared samples. As the photons moves through a thicker sample, they encounter more atoms and potential scatterers within the sample, which increased the interaction with the thicker sample results in more photons being attenuated, thereby decreasing the TF^39^. Furthermore, both samples with multilayer’s have lower TF than the samples with only one layer (see Fig. 10). This highlights the significance of developing radiation shielding materials with multilayer structures, as the multilayer samples exhibit superior attenuation effectiveness when compared to the samples with single layers.

From this result, we can summarize the following; Utilizing multilayer components, which are superior to single-layer ones since each layer can provide distinct attenuation qualities due to its distinct density, thickness, or atomic composition, resulting in more varied interactions with the radiation, can enhance the efficiency of radiation protection. A multilayer material can also have its layers ordered in complimentary ways, which improves the material's capacity for radiation absorption and scattering and makes it more efficient than a single-layer material with an average atomic number.

**Figure 11:**
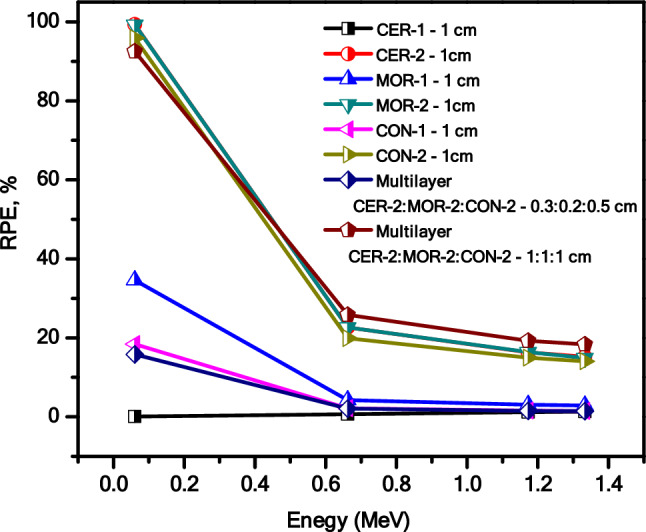
The RSE of all prepared composites as well as different multi-layers at different energies.

In addition to this, we examined at the RPE of both the prepared materials and the materials that had three layers (Fig. 11). It is clear to see that the Con-2 sample with a thickness of 1 cm has extremely low RPE values (it is 16% at 0.06 MeV and in the range of 1–2% at the other energies). This is because this sample possesses high TF. This indicates that the radiation attenuation effectiveness of this sample is weak, and the majority of photons can pass easily through it. It can prevent a small amount of radiation with energy of 0.06 MeV, but the majority of radiation with energy of 0.662 to 1.333 MeV can easily pass through this sample.

The same result is obtained for Mo-10%, where the RPE is only 18% at 0.06 MeV and in the range of 1–2% at the other energies. Cer-2 has an RPE of 34% at 0.06 MeV, but at other energies, it remains in the range of 2–4%. This indicates that Cer-2 is a more effective attenuator than Con-2 and Mor-2. When we examine the RPE for samples that include 30% HOM nanoparticles, we find that it is approximately 100% at 0.06 MeV, indicating that these samples are excellent attenuators because of the significant amount of HMO present in them. This once more demonstrates the significance of employing HMO to improve the samples' radiation shielding capabilities. This addition of HMO increases the probability of the interaction between the photons and the sample, which improves the radiation shielding properties^40^. The RPE for these specimens at 0.662 MeV is in the range of 19–22%, however, and as a result, these materials exhibit poor radiation shielding ability for radiation with energies higher than 0.662 MeV.

Upon examining the RPE of the multilayer samples at 0.662 MeV, it was noticed that the sample comprising Cer-2, Mor-2, and Con-2, with thicknesses of 0.2 cm, 0.3 cm, and 0.5 cm, had an RPE of 95%. This indicates that this sample exhibits excellent attenuation performance. However, at 1.173 MeV and 1.333 MeV, the RPE was 90% and 89%, respectively. This suggests that the sample with three layers, each with a thickness of 1 cm, is a suitable attenuator for radiation with an energy range of 0.06–1.333 MeV. Overall, these findings demonstrate the effectiveness of the multilayer samples in attenuating radiation across a range of energies.

**Figure 12:**
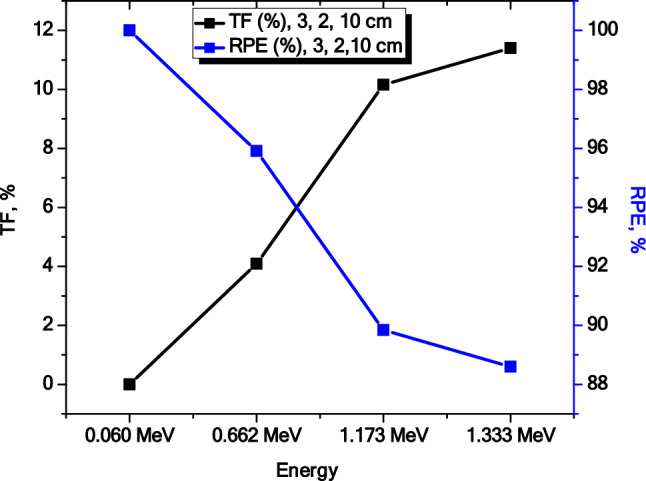
The TF and RSE of multilayer’s of Cer-2: Mor-2: Con-2 at different energies.

Our analysis involved plotting the TF and RPE values against energy (see Fig. 12). Interestingly, these two parameters showed opposite trends with respect to energy. It’s worth noting that the sum of RPE and TF is always equal to 100%. For instance, at 0.06 MeV, RPE was 88%, while TF was 12%. This implies that RPE reflects the effectiveness of the sample in shielding radiation, while TF reveals the number of photons that can penetrate the material. Since that both RPE and TF are crucial considerations for evaluating the efficacy of materials in radiation shielding purposes, this complementing information is useful. Our findings suggest that by enhancing these variables, radiation shielding materials for a variety of applications might be developed that are more effective and efficient.

## Conclusion

In conclusion, this study explored the development of multilayer radiation shielding systems using ceramics, concrete, and mortar, incorporating Bi_2_O_3_, WO_3_, and PbO nanoparticles to optimize photon (X- and gamma-ray) attenuation. Samples with varying thicknesses and concentrations of nanoparticles were prepared and analyzed using experimental methods. The SEM and EDX analyses provided insights into the elemental composition and particle distribution of the samples, highlighting the superior distribution of ceramic particles and reduced voids compared to mortar and concrete. The linear attenuation coefficient (LAC) evaluation, conducted through experimental measurements and Phy-X software calculations, demonstrated a strong correlation between the two methods. Samples containing 30% heavy metal oxides exhibited higher LAC values than those with 10%, emphasizing the importance of higher oxide concentrations for better attenuation properties. Among the three materials, ceramics were found to be the most effective in terms of LAC values. We developed multiple layers with thicknesses of 2 and 3 cm and we found that the TF of the sample with a multilayer and a thickness of 3 cm is lower than the TF of the sample with the same multilayer and a thickness of 2 cm. The results demonstrated that utilizing multilayer components can provide distinct attenuation qualities due to its distinct density, thickness, or atomic composition, resulting in more varied interactions with the radiation, can enhance the efficiency of radiation protection.

## Data Availability

All data generated or analyzed during this study are included in this published article.
